# Fragile self, mechanical world: mechanistic delusions and ego fragility in schizotypal–affective spectrum disorder—a CARE case report

**DOI:** 10.3389/fpsyt.2026.1896591

**Published:** 2026-07-16

**Authors:** Giovanna Azevedo Rodrigues, Fernando Martins Castanheira Junior, Larissa Leite Vieira de Oliveira, Marcelo Caixeta

**Affiliations:** Department of Psychiatry, Associação de Saúde Mental e Infantil de Goiás (ASMIGO), Goiania, Brazil

**Keywords:** case report, ego fragility, limbic hyperactivation, mechanistic delusions, mirror neuron system, psychopathology, relational causality, schizotypal-affective spectrum

## Abstract

Categorical nosological systems frequently fall short when confronted with patients whose presentations cross established diagnostic boundaries. We report M.S., a 44-year-old Brazilian man involuntarily admitted to a psychiatric inpatient unit who presented with systematized persecutory ideation of mechanistic–technological content (chip implantation, satellite-based surveillance), structural ego fragility, absent insight, and progressive social and occupational deterioration. While prior diagnoses of bipolar disorder and provisional schizophrenia had been considered, neither fully captured the clinical complexity. Psychopathological-dimensional analysis, grounded in phenomenological observation and contemporary psychopathological theory, suggests three potentially interacting axes: (1) structural ego fragility (Ich-Schwäche), potentially arising from impaired early attachment and deficient relational learning; (2) a relational causality deficit replaced by concrete–mechanistic reasoning; and (3) limbic hyperactivation that appears to sustain an anxiety–perplexity–paranoia feedback loop. These converge on a schizotypal–affective spectrum formulation. Laboratory investigations identified severe dyslipidemia and marked hyperandrogenism (total testosterone 1,367 ng/dL), the latter potentially associated with limbic hyperactivation, though causality cannot be established from a single cross-sectional measurement. Psychometric assessment (BPRS-18) at admission yielded a total score of 43, with suspiciousness (5) and unusual thought content (5) as dominant items. During a seven-day inpatient course, a multimodal thymic strategy—risperidone, lithium carbonate, and structured psychotherapy—produced attenuation of paranoid reactivity, improved family engagement, and the spontaneous resumption of guitar playing from day 3, as a functional correlate of behavioral stabilization. The patient was discharged with a referral for a three-monthly paliperidone palmitate long-acting injectable. The pharmacological response retrospectively supports the dimensional formulation and illustrates the heuristic value of psychopathological analysis grounded in ego structure, causal reasoning, and affective dysregulation as a complementary approach to categorical nosology in complex psychotic–affective presentations.

## 1 Introduction

The history of psychiatric nosology is marked by persistent tension between the utility of categorical diagnoses and the clinical reality of patients who defy rigid classification boundaries. Both DSM-5-TR and ICD-11 have progressively incorporated dimensional spectra, yet clinicians regularly encounter presentations whose phenomenological richness surpasses any single diagnostic label (1, 2). Patients exhibiting chronic persecutory ideation with bizarre content, preserved cognitive functioning, and features of both personality disturbance and affective dysregulation constitute a particularly demanding subgroup. Standard differential categories—schizophrenia, schizoaffective disorder, bipolar disorder with psychotic features, delusional disorder, and schizotypal personality disorder—often yield inconclusive or contradictory results (3, 4).

A psychopathological approach grounded in psychic structure, the ontogeny of the self, and the phenomenology of thought offers an alternative framework rooted in classical psychopathology (Jaspers, Bleuler, Minkowski) and enriched by contemporary neurobiological concepts (5–8). We present a case in which this dimensional model clarifies a presentation resisting categorical nosological placement and informs a precisely targeted treatment strategy.

## 2 Patient information

### 2.1 Patient demographics and anonymization

The patient is a 44-year-old Brazilian man, unmarried, living with his family. Prior to the onset of progressive functional decline, he was employed in technical and musical domains; at the time of admission, he was unemployed. In accordance with CARE guidelines, all identifying information has been removed or anonymized to protect patient confidentiality.

### 2.2 Presenting complaint

Chief complaint: “I don’t know why they brought me here. My family is hiding things from me.” The family reported progressive behavioral deterioration, intense irritability, systematized persecutory beliefs, and repeated treatment discontinuation. On initial contact, the patient oscillated between partial acknowledgment and firm denial of persecution; his older brother described the orthopedic fixation pin in his knee as a suspected locus of external control.

## 3 Clinical findings

### 3.1 General physical and neurological examination

On admission, the patient was normolipemic, anicteric, and acyanotic. Vital signs revealed persistent arterial hypertension (blood pressure consistently 150–170 mmHg/100–120 mmHg). Cardiac and pulmonary auscultation were unremarkable. The abdomen was soft, non-tender, with normal bowel sounds. A prominent orthopedic fixation pin was noted in the left knee, secondary to a previous anterior cruciate ligament (ACL) reconstruction. Neurological examination revealed no focal motor or sensory deficits; cranial nerves were intact, deep tendon reflexes were symmetric (2+), and cerebellar function was preserved. No extrapyramidal signs were present at baseline.

### 3.2 Mental status examination

On admission, the patient was cooperative and initially cordial. Speech was fluent, prolix, and tangential. Thought content was dominated by systematized persecutory beliefs directed at family members, neighbors, and professional competitors, organized around an implicit grandiose premise—namely, above-average talent in music and technical domains provoking envy and deliberate sabotage. Ideas of reference with technological-mechanistic content were particularly salient: electronic surveillance drones and concealed cameras monitored his home; his brother (an IT professional) could remotely access his devices; and an orthopedic fixation pin might function as an implanted control instrument. These beliefs were held with partial conviction. Affect oscillated between expansiveness and irritability; critical insight was severely compromised; no hallucinations were elicited; no suicidal or heteroaggressive ideation was identified.

### 3.3 History of present illness

Parental accounts document an atypical cognitive profile from early childhood: intense preoccupation with outer space, extraterrestrial life, mechanics, and technology. Polysubstance use commenced between the ages of 13 and 16 (cannabis, cocaine, crack cocaine, inhalants, alcohol, tobacco), motivated not by hedonism but by a wish to “expand consciousness” and “see beyond ordinary reality”—reflecting an already-porous schizotypal boundary between internal and external reality. Peak use extended until ages 28–29, followed by sustained illicit-drug abstinence. A first psychiatric evaluation in early adulthood yielded diagnoses of bipolar disorder and provisional schizophrenia; a prior hospitalization (approximately 5 years before) was experienced as coercive. In the years before the current admission, the patient exhibited progressive occupational decline, verbal aggression toward the family, one police intervention, and object hoarding. Psychotropic treatment had been abandoned entirely, with the police retaining only zolpidem 10 mg.

### 3.4 Psychiatric and medical history

The best pharmacological response on record was to quetiapine 100 mg. The patient suicidal ideation approximately 15 years earlier, but no suicide attempts. The patient underwent ACL reconstruction (left knee) following a motorcycle accident; the fixation pin was subsequently incorporated into the delusional nucleus. Blood pressure was consistently 150–170 mmHg/100–120 mmHg throughout admission, indicating previously unrecognized arterial hypertension. A first-degree cousin carries a probable schizophrenia diagnosis; an uncle has a probable psychotic disorder.

### 3.5 Laboratory investigations

Comprehensive investigations were performed on 19 May 2026. Routine parameters (hematological, renal, hepatic, glycemic, thyroid, and infectious serologies) were within normal limits. Clinically significant abnormalities included severe mixed dyslipidemia (total cholesterol 427, LDL 339, triglycerides 168 mg/dL); homocysteine at the upper limit of normal; and suboptimal vitamin D (**Table 1**).

**Table 1:** Selected laboratory results (19 May 2026).

Parameter	Result	Reference range
Total cholesterol	**427 mg/dL**	<190 mg/dL
LDL cholesterol	**339 mg/dL**	<130 mg/dL
Triglycerides	**168 mg/dL**	<150 mg/dL
HDL cholesterol	54 mg/dL	>40 mg/dL
Total testosterone	**1,367 ng/dL**	300 ng/dL–1,000 ng/dL
SHBG	86.2 nmol/L	24.3 nmol/L–122 nmol/L
Homocysteine	14.8 µmol/L	5.0 µmol/L–15.0 µmol/L
Vitamin D (25-OH)	**28 ng/mL**	30 ng/mL–100 ng/mL
Fasting glucose	88 mg/dL	70 mg/dL–99 mg/dL
TSH	1.8 µIU/mL	0.4 µIU/mL–4.0 µIU/mL
AST/ALT	22/28 U/L	<40 U/L/<41 U/L

Clinically significant abnormalities highlighted in bold.

#### 3.5.1 Endocrinological findings—limitations and interpretation

Of particular endocrinological interest, total testosterone was markedly elevated at 1,367 ng/dL with a normal SHBG level of 86.2 nmol/L, indicating genuine hyperandrogenism. However, several important caveats apply to the interpretation of this finding. First, testosterone was measured once during acute psychiatric admission; acute stress, sleep deprivation, and acute psychotic symptoms are known to alter testosterone levels, and this single cross-sectional measurement may reflect state-dependent changes rather than a stable trait. Second, a complete endocrine evaluation was not performed. We did not measure LH, FSH, or prolactin, nor did we conduct imaging of the pituitary or adrenal glands. Without these data, the etiology of the hyperandrogenism (primary testicular, secondary to pituitary or hypothalamic dysfunction, or adrenal) cannot be determined. Third, given the patient’s history of polysubstance use, the possibility of exogenous anabolic steroid use could not be definitively excluded, representing a significant limitation in the clinical workup. Fourth, while the patient had been off psychotropic medications for approximately one year prior to admission, his history of prior antipsychotic exposure (e.g., quetiapine) may have residual effects on gonadal function. Finally, while hyperandrogenism could theoretically contribute to irritability, affective dysregulation, and paranoid reactivity, the temporal relationship between the testosterone elevation and psychotic symptom onset remains unknown. Consequently, while the hyperandrogenism is a noteworthy biological finding, a causal relationship with the patient’s psychopathology cannot be established from the available data.

### 3.6 Psychometric assessment (BPRS-18)

The assessment was administered on 18 May 2026. The total score was 43 (range, 18–126; moderate severity). Dominant items were suspiciousness (5), unusual thought content (5), tension (4), anxiety (4), grandiosity (4), and hostility (4). Near-absent hallucinatory behavior (1), motor retardation (0), and guilt (0), together with preserved orientation, were consistent with a schizotypal-spectrum rather than classical schizophrenia presentation.

## 4 Clinical timeline

The longitudinal course encompassed the following: childhood idiosyncratic cognitive profile; polysubstance use between the ages of 13 and 29; first psychiatric evaluation and bipolar/schizophrenia diagnoses in young adulthood; first inpatient admission approximately in 2021 (best response: quetiapine 100 mg); progressive deterioration from 2021 to 2025 (job loss, family conflict, object hoarding, treatment abandonment); and current involuntary admission in 2026.

## 5 Diagnostic assessment

### 5.1 The limits of categorical diagnosis

A thorough review of the case highlights the challenges of applying single categorical labels to complex presentations at the psychotic–affective-personality spectrum boundary. While the patient’s presentation does not fully satisfy DSM-5-TR criteria for classical schizophrenia—given his intact cognitive function, preserved prior occupational competence, and absence of prominent negative symptoms or formal thought disorder—a diagnosis of schizophrenia spectrum disorder remains clinically plausible given the chronic persecutory ideation, functional decline, and positive family history. Similarly, while the continuous nature of his psychotic ideation makes bipolar disorder with psychotic features less parsimonious, bipolar spectrum presentations with persistent psychotic features between mood episodes have been documented in the literature and remain a reasonable alternative formulation. Schizoaffective disorder also remains diagnostically plausible if one interprets the patient’s affective instability and irritability as meeting the threshold for a major mood episode. Delusional disorder is less likely given that the beliefs are explicitly bizarre (chip implantation, remote control) and span multiple domains.

The value of the dimensional formulation lies not in excluding these categorical diagnoses, but in identifying specific psychopathological mechanisms that may guide targeted treatment. A patient meeting criteria for a schizophrenia spectrum disorder can simultaneously be understood through the lens of ego fragility and relational causality deficit. The schizotypal–affective spectrum formulation offers additional clinical utility by organizing the patient’s presentation around core psychopathological dimensions—ego structure, concrete–mechanistic reasoning, affective instability, and bizarre ideation—that cut across categorical boundaries and inform a precise therapeutic strategy.

### 5.2 Psychopathological formulation: four interacting axes

The pivotal psychopathological finding in this case is the structural weakness of the “secondary self” (Ich-Schwäche): the reflexive and metacognitive layer of identity that sustains self-observation, reality testing, and affective regulation (7, 9).

The formulation rests on four proposed axes:

AXIS I (Structural): Ego fragility (Ich-Schwäche), potentially arising from impaired early attachment, contributing to identity diffusion, stimulus hypersensitivity, and absent insight.
AXIS II (Cognitive): Relational causality deficit, potentially mediated by mirror neuron system impairment, whereby concrete–mechanistic formal reasoning replaces interpersonal social inference, yielding bizarre yet formally coherent delusional content.
AXIS III (Affective): Endogenous thymic dysregulation and limbic hyperactivation, which appear to sustain a primary anxiety → perplexity → paranoia → delusional elaboration feedback loop.
AXIS IV (Developmental): Early adversity and polysubstance use (ages 13–29), which may potentiate Axes I–III and lower the threshold for psychotic elaboration.

## 6 Therapeutic interventions

### 6.1 Pharmacological strategy

Treatment was guided by the dimensional formulation. The primary antipsychotic selected was risperidone at 1.5 mg/day (later increased to 2.5 mg/day), chosen for its dopamine antagonism and affective stabilization properties. Haloperidol 15 mg nocte and levomepromazine 25 mg nocte were used temporarily to manage acute agitation and paranoid reactivity. Lithium carbonate 375 mg/day was added to address bipolar-spectrum affective instability and to potentiate antipsychotic efficacy. Losartan 50 mg/day (an angiotensin receptor blocker) was initiated to manage previously unrecognized arterial hypertension. The transition to three-monthly intramuscular paliperidone palmitate 100 mg was planned for discharge to improve medication adherence, reduce the risk of relapse, and provide sustained dopamine antagonism.

#### 6.1.1 Rationale for antipsychotic selection and monitoring

Risperidone was selected over other agents given because of its balanced dopamine and serotonin antagonism, which may address both positive symptoms and affective dysregulation. Baseline extrapyramidal assessment was performed; no extrapyramidal signs were present at baseline. Haloperidol and levomepromazine were used as temporary adjuncts during acute crisis stabilization but were not intended as long-term agents. The transition to paliperidone palmitate provides a long-acting formulation that may enhance adherence and clinical stability in a patient with significant insight impairment and prior treatment discontinuation.

### 6.2 Psychotherapeutic approach

Structured psychotherapy was implemented alongside pharmacological intervention, focusing on (1) psychoeducation regarding the patient’s psychiatric condition and the rationale for treatment; (2) reality testing and gentle exploration of the evidence for and against persecutory beliefs; (3) engagement with family members to improve understanding and reduce expressed emotion; and (4) identification of functional goals and reinforcement of adaptive behaviors. The therapeutic approach was informed by principles of motivational interviewing and cognitive remediation, adapted to the patient’s limited insight and concrete cognitive style.

## 7 Clinical course and outcomes

### 7.1 Inpatient clinical course

Day 1 (Admission): The patient was admitted involuntarily following a family request and police involvement. Initial presentation: agitated, suspicious, tangential speech, systematized persecutory beliefs, poor insight. BPRS-18 score: 43. Risperidone 1.5 mg/day initiated; haloperidol 15 mg nocte and levomepromazine 25 mg nocte added for acute agitation. Lithium carbonate 375 mg/day initiated. Losartan 50 mg/day initiated for hypertension management.
Day 2: Patient remained guarded but cooperative with nursing staff. Paranoid ideation remained unchanged. Risperidone was increased to 2.5 mg/day. A family meeting was held to provide psychoeducation and assess social support.
Day 3: The spontaneous resumption of guitar playing was noted—first functional improvement. The patient engaged in ward activities. Paranoid reactivity began to attenuate. The family reported improved communication.
Day 4–5: Progressive attenuation of paranoid reactivity was observed. The patient engaged more openly in psychotherapy sessions. Reality-testing discussions were initiated. No adverse effects of the medications were noted.
Day 6: Marked improvement in affect and social engagement was observed. The patient demonstrated temporary restoration of secondary-self functions and the emergence of a rudimentary therapeutic alliance. Discharge planning and referral for a long-acting injectable were discussed.
Day 7 (Discharge): The patient discharged with prescriptions for risperidone 2.5 mg/day, lithium carbonate 375 mg/day, and losartan 50 mg/day. A referral was made for three-monthly intramuscular paliperidone palmitate 100 mg. Outpatient psychiatry follow-up was scheduled. The family was provided with written psychoeducational materials and crisis contact information.

## 8 Discussion

### 8.1 Clinical significance of the case

This case illustrates the clinical utility of dimensional psychopathological analysis in complex presentations that resist categorical nosological placement. The patient’s combination of chronic persecutory ideation with bizarre technological–mechanistic content, preserved cognitive function, structural ego fragility, and affective dysregulation does not fit neatly into any single DSM-5-TR or ICD-11 category. The schizotypal–affective spectrum formulation, grounded in ego structure, relational causality deficit, and limbic hyperactivation, provides a more nuanced understanding of the underlying psychopathology and informs a precisely targeted treatment strategy.

### 8.2 Psychopathological mechanisms

The four-axis formulation proposes that this patient’s presentation results from the convergence of multiple psychopathological mechanisms. Structural ego fragility (Ich-Schwäche), potentially arising from impaired early attachment and deficient relational learning, contributes to identity diffusion, stimulus hypersensitivity, and absent insight. A relational causality deficit, potentially mediated by mirror neuron system impairment, results in the replacement of interpersonal social inference with concrete–mechanistic formal reasoning, yielding bizarre yet formally coherent delusional content. Endogenous thymic dysregulation and limbic hyperactivation appear to sustain a feedback loop from anxiety to perplexity to paranoia to delusional elaboration. Early adversity and polysubstance use (ages 13–29) may potentiate these three axes and lower the threshold for psychotic elaboration.

### 8.3 Mirror neuron system and relational causality deficit

The mirror neuron system (MNS) is a network of neurons that fire both when an individual performs an action and when they observe another individual performing the same action. Dysfunction of the MNS has been hypothesized to contribute to deficits in social understanding, empathy, and theory of mind in various psychiatric conditions (10–12). In this case, we propose that MNS impairment may underlie the patient’s relational causality deficit—the inability to infer the mental states and intentions of others and to attribute causality to interpersonal factors. Instead, the patient resorts to concrete–mechanistic reasoning, interpreting social interactions through the lens of technological control and external manipulation. In particular, the MNS is utilized here as a heuristic psychopathological model to explain this causality deficit, rather than as a proven neuroimaging biomarker for this specific patient. This model is inferred from clinical observation rather than being directly measured through neuroimaging or electrophysiological assessment.

### 8.4 Biological findings and their interpretation

The marked hyperandrogenism (total testosterone 1,367 ng/dL) is a noteworthy biological finding. Elevated androgens have been associated with increased irritability, affective dysregulation, and paranoid reactivity in some studies (13, 14). However, as discussed in *Section 3.5.1*, this finding must be interpreted with considerable caution. The testosterone measurement was performed once during acute psychiatric admission, when stress, sleep deprivation, and acute psychotic symptoms are known to alter hormone levels. A complete endocrine evaluation was not performed, and the etiology of the hyperandrogenism cannot be determined. The possibility of exogenous anabolic steroid use cannot be definitively excluded. Consequently, while the hyperandrogenism is a noteworthy biological finding, a causal relationship with the patient’s psychopathology cannot be established from the available data.

### 8.5 Limitations

This case report has several important limitations. First, the assessment of ego structure and relational causality deficit relies on clinical observation and psychometric testing (BPRS-18) rather than on objective neurobiological measures. Second, the BPRS-18 has known limitations in capturing the full complexity of psychopathology and may not be sensitive to subtle changes in ego structure or relational reasoning. Third, the historical information regarding early attachment and relational learning is based on parental recollection and may be subject to bias. Fourth, no neuroimaging studies (MRI, PET, fMRI) were performed, limiting our ability to assess structural or functional brain abnormalities. Fifth, the mirror neuron system model is speculative and was not directly tested in this case. Sixth, the follow-up period is limited to a single 7-day inpatient hospitalization, and longer-term outcomes are unknown. Seventh, the marked hyperandrogenism was not fully investigated, and the etiology remains unclear. Eighth, this is a single case report, and the findings cannot be generalized to other patients with similar presentations.

### 8.6 Clinical implications

Despite these limitations, this case illustrates several important clinical principles. First, dimensional psychopathological analysis can provide valuable clinical utility in complex presentations that resist categorical nosological placement. Second, a precise understanding of underlying psychopathological mechanisms can guide targeted treatment strategies. Third, multimodal interventions (pharmacological and psychotherapeutic) may be more effective than monotherapy in addressing complex presentations. Fourth, the pharmacological response to risperidone, lithium, and structured psychotherapy provides retrospective support for the dimensional formulation and suggests that the proposed psychopathological mechanisms may have clinical validity.

## 9 Conclusion

We present a case of a 44-year-old man with a complex psychiatric presentation characterized by chronic persecutory ideation with bizarre technological–mechanistic content, structural ego fragility, and affective dysregulation. The patient’s presentation does not fit neatly into any single categorical diagnosis but is well-captured by a schizotypal–affective spectrum formulation grounded in ego structure, relational causality deficit, and limbic hyperactivation. A multimodal treatment strategy—risperidone, lithium carbonate, and structured psychotherapy—produced significant clinical improvement, with attenuation of paranoid reactivity, improved family engagement, and resumption of functional activities. The case illustrates the heuristic value of psychopathological analysis grounded in ego structure, causal reasoning, and affective dysregulation as a complementary approach to categorical nosology in complex psychotic–affective presentations. Future research should investigate the neurobiological basis of ego fragility, relational causality deficit, and mirror neuron system dysfunction in schizotypal–affective spectrum presentations.

## Data Availability

The original contributions presented in the study are included in the article/**Supplementary Material**. Further inquiries can be directed to the corresponding author.
