# Supplementary material for: Fragile self, mechanical world: mechanistic delusions and ego fragility in schizotypal–affective spectrum disorder—a CARE case report
Source: Front Psychiatry. 2026 Jul 16;17:1896591. doi: 10.3389/fpsyt.2026.1896591 (PMC13422387; doi:10.3389/fpsyt.2026.1896591)
Supplement: Supplementary file 1 [file SupplementaryFile1.docx]

**CARE Checklist (2013)**

*Information to include when writing a case report*

*Manuscript: "Fragile Self, Mechanical World: Mechanistic Delusions and Ego Fragility in Schizotypal-Affective Spectrum Disorder — A CARE Case Report" | Frontiers in Psychiatry — Schizophrenia | 2026*

| **TOPIC** | **ITEM #** | **CHECKLIST ITEM DESCRIPTION** | **REPORTED ON (SECTION)** | **PRESENT?** |
| --- | --- | --- | --- | --- |
| **Title** | 1 | The words "case report" should be in the title along with what is of greatest interest in this case. | *Title* | Yes  Title: "…Schizotypal-Affective Spectrum Disorder — A CARE Case Report" |
| **Key Words** | 2 | The key elements of this case in 2 to 5 key words (including "case report"). | *Title/Key Words* | Yes  8 keywords including "case report", "schizotypal-affective spectrum", "ego fragility" |
| **Abstract** | 3a | Introduction — What is unique about this case? What does it add to the medical literature? | *Abstract* | Yes  Presentation crossing psychotic-affective-personality spectrum boundaries; inadequacy of standard DSM categories; four-axis dimensional model |
|  | 3b | The main symptoms of the patient and the important clinical findings. | *Abstract* | Yes  Systematised mechanistic-technological persecutory ideation; structural ego fragility; absent insight; BPRS-18 total 43 |
|  | 3c | The main diagnoses, therapeutics interventions, and outcomes. | *Abstract* | Yes  Schizotypal-affective spectrum formulation; multimodal thymic strategy (haloperidol decanoate, lithium, psychotherapy); discharge day 7 with improved insight |
|  | 3d | Conclusion — What are the main "take-away" lessons from this case? | *Abstract* | Yes  Dimensional psychopathological analysis superior to categorical nosology for spectrum-boundary presentations |
| **Introduction** | 4 | Brief background summary of this case referencing the relevant medical literature. | *Introduction (Sec. 1)* | Yes  Limits of DSM-5-TR/ICD-11 categorical models; mirror neuron system; ego structure framework; refs [1–8] |
| **Patient Information** | 5a | Demographic information (age, gender, ethnicity, occupation). | *Patient Information (Sec. 2, Table)* | Yes  M.S., 44-year-old male; music teacher; university graduate; single; Evangelical Christian; data anonymised |
|  | 5b | Main symptoms of the patient (chief complaints). | *Patient Information (Sec. 2)* | Yes  “I don’t know why they brought me here” — persecutory beliefs, irritability, treatment discontinuation |
|  | 5c | Medical, family, and psychosocial history including co-morbidities and relevant genetic information. | *Patient Information (Sec. 3.2–3.4)* | Yes  Polysubstance use age 13–29; prior hospitalisation; ACL reconstruction; family hx schizophrenia (cousin); absent hypertension awareness; quetiapine prior best response |
|  | 5d | Relevant past interventions with outcomes. | *Patient Information (Sec. 3.3)* | Yes  Quetiapine 100 mg — best pharmacological response on record; prior admission experienced as coercive; medication discontinuation history |
| **Clinical Findings** | 6 | Describe significant physical examination and important clinical findings. | *Clinical Findings (Sec. 3.1, 3.4–3.5)* | Yes  MSE: persecutory-mechanistic ideation, grandiose-persecutory dyad, partial conviction, absent insight; BP 150–170/100–120 mmHg; BPRS-18 = 43; labs: total cholesterol 427, testosterone 1,367 ng/dL |
| **Timeline** | 7 | Historical and current information from this episode of care organised as a timeline (figure or table). | *Timeline (Sec. 4, Figure 2)* | Yes  Sec. 4 + Figure 2 (CARE-compliant timeline from birth 1981 to discharge 25 May 2026, 7 parallel tracks) |
| **Diagnostic Assessment** | 8 | Diagnostic methods (PE, laboratory testing, imaging, questionnaires). | *Diagnostic Assessment (Sec. 3.4–3.5, Sec. 5)* | Yes  BPRS-18 (day 1); full metabolic/endocrine panel (Table 1); psychopathological-dimensional analysis; systematic evaluation of 4 categorical diagnoses |
|  | 9 | Diagnostic reasoning including other diagnoses considered. | *Diagnostic Assessment (Sec. 5.1)* | Yes  Schizophrenia, bipolar disorder, schizoaffective disorder, delusional disorder systematically excluded; schizotypal-affective spectrum formulation — best fit |
| **Therapeutic Interventions** | 10a | Types of intervention (pharmacological, surgical, preventive, self-care). | *Therapeutic Interventions (Sec. 6)* | Yes  Risperidone 1.5→2.5 mg/day; haloperidol decanoate 100 mg IM; lithium carbonate 37.5→375 mg; levomepromazine 25 mg; zolpidem 10 mg; structured psychotherapy; family psychoeducation |
|  | 10b | Administration of intervention (dosage, strength, duration). | *Therapeutic Interventions (Sec. 6.1, Sec. 7.1)* | Yes  Full day-by-day pharmacological regimen documented in Sec. 7.1; dosages and titrations specified |
|  | 10c | Intervention adherence and tolerability (how was this assessed?). | *Therapeutic Interventions (Sec. 7.1)* | Yes  Acute dystonia (day 3) resolved with IV biperiden 5 mg; subsequent full acceptance of all injectables; clinical observation throughout admission |
|  | 10d | Adverse and unanticipated events. | *Therapeutic Interventions (Sec. 7.1)* | Yes  Acute dystonia after haloperidol decanoate 100 mg IM; resolved IV biperiden; hypertension (150–170 mmHg) requiring ARB; persistent insomnia requiring hypnotic escalation |
| **Discussion** | 11a | Strengths and limitations in the management of this case. | *Discussion (Sec. 8.5)* | Yes  Sec. 8.5 lists 7 explicit limitations (residency setting, instruments, informant bias, no neuroimaging, MNS speculation, 7-day follow-up, hyperandrogenism uninvestigated) |
|  | 11b | Discussion of the relevant medical literature. | *Discussion (Sec. 8.1–8.4)* | Yes  RDoC, DTI meta-analysis [30], schizotypy 8-yr follow-up [31], MNS meta-analysis [32], neuroinflammation [33–34], Brazilian psychiatric reform [36–38] |
|  | 11c | The rationale for conclusions (including assessment of possible causes). | *Discussion (Sec. 8.2–8.4)* | Yes  Pharmacological response retrospectively validates dimensional formulation; thymic strategy response confirms schizotypal-affective rather than purely schizophrenic or bipolar substratum |
|  | 11d | The main "take-away" lessons of this case report. | *Discussion/Conclusion (Sec. 9)* | Yes  4 principal conclusions (Sec. 9): ego fragility as primary substratum; delusional content mirrors pre-morbid cognitive style; limbic loop perpetuates phenomenology; treatment must address all 3 levels simultaneously |
| **Patient Perspective** | 12 | Did the patient share their perspective or experience? (Include when appropriate.) | *Patient Perspective (box)* | Yes  “I really liked that injection — I feel much better now.” Day 6: resumed guitar playing; requested to give music lessons; agreed to reside near mother post-discharge |
| **Informed Consent** | 13 | Did the patient give informed consent? Please provide if requested. | *Consent box / Declarations* | Yes  Written informed consent obtained in accordance with Declaration of Helsinki (2013 revision). Copy available for Editor-in-Chief upon request. |

CARE Checklist (2013) — Riley DS et al. Guidelines for writing a case report. Hosp Pediatr. 2017;7(7):415–419. doi: 10.1542/hpeds.2017-0017 | Gagnier JJ et al. J Clin Epidemiol. 2013;66(11):1263–1268. doi: 10.1016/j.jclinepi.2013.06.002 | www.care-statement.org

*Authors: Rodrigues GA, Castanheira F, Oliveira LLV, Caixeta M | Completed: 31 May 2026 | Submitted to: Frontiers in Psychiatry — Schizophrenia*
